# *In-vivo* detection of binary PKA network interactions upon activation of endogenous GPCRs

**DOI:** 10.1038/srep11133

**Published:** 2015-06-23

**Authors:** Ruth Röck, Verena Bachmann, Hyo-eun C Bhang, Mohan Malleshaiah, Philipp Raffeiner, Johanna E Mayrhofer, Philipp M Tschaikner, Klaus Bister, Pia Aanstad, Martin G Pomper, Stephen W Michnick, Eduard Stefan

**Affiliations:** 1 Institute of Biochemistry and Center for Molecular Biosciences, University of Innsbruck, Innrain 80/82, 6020 Innsbruck, Austria; 2 Russell H. Morgan Department of Radiology and Radiological Science, Johns Hopkins Medical School, Baltimore, MD 21287, USA; 3 Département de Biochimie, Université de Montréal, H3C 3J7 Montréal, Québec, Canada; 4 Institute of Molecular Biology, University of Innsbruck, Technikerstrasse 25, 6020 Innsbruck, Austria

## Abstract

Membrane receptor-sensed input signals affect and modulate intracellular protein-protein interactions (PPIs). Consequent changes occur to the compositions of protein complexes, protein localization and intermolecular binding affinities. Alterations of compartmentalized PPIs emanating from certain deregulated kinases are implicated in the manifestation of diseases such as cancer. Here we describe the application of a genetically encoded Protein-fragment Complementation Assay (PCA) based on the *Renilla* Luciferase (*R*luc) enzyme to compare binary PPIs of the spatially and temporally controlled protein kinase A (PKA) network in diverse eukaryotic model systems. The simplicity and sensitivity of this cell-based reporter allows for real-time recordings of mutually exclusive PPIs of PKA upon activation of selected endogenous G protein-coupled receptors (GPCRs) in cancer cells, xenografts of mice, budding yeast, and zebrafish embryos. This extends the application spectrum of *R*luc PCA for the quantification of PPI-based receptor-effector relationships in physiological and pathological model systems.

Most signal transduction pathways transmit receptor-mediated input signals through a relay of intracellular signaling events. Signals are sensed and converted by defined cell surface receptors and are transmitted through cytoplasmic and nuclear effector molecules. This leads to signal amplification and uncoupling as well as feed-forward and feed-back regulation, which are based amongst others on oscillations of second messenger levels and on modulations of dynamic protein-protein interactions (PPIs). These PPIs connect signaling cascades and participate in the co-ordination of the plethora of extracellular stimuli to convert them into physiological but also pathological responses within cells^1^,^2^. Deregulated upstream signaling and mutations in kinases and their regulatory proteins alter spatially controlled PPIs which account for kinases-induced carcinogenesis^3^,^4^,^5^,^6^,^7^. One of the best studied example for an evolutionarily conserved small molecule sensor and dynamic PPI is the cAMP-dependent protein kinase A (PKA). PKA acts as a compartmentalized signaling hub of multiple signaling cascades and is therefore a central regulator and effector of homeostatic and metabolic control^8^. It is the classic downstream effector of G protein-coupled receptors (GPCRs) which are among the most common targets of therapeutic drug development^9^,^10^,^11^. PKA is targeted to diverse subcellular locations through PPIs with diverse A-kinase anchor proteins (AKAPs), which is a precondition for spatially and temporally controlled PKA-substrate phosphorylation^8^,^12^. Activation of compartmentalized PKA pools is dependent on direct binding of the second messenger cAMP to PKA regulatory subunits type I (RI) or II (RII). cAMP binding triggers dissociation and activation of PKA catalytic subunits (PKAc). Nuclear PKAc subunits are specifically inhibited through the protein kinase inhibitor peptide (PKI), which binds to and exports PKAc from the nucleus^13^,^14^,^15^. Deregulation of cAMP/PKA functions contribute to the manifestation of diseases such as cancer. Distinct R or PKAc kinase gene mutations/fusions provoke permanent PKAc activation which participate in disease etiology and progression. As examples, mutations in RIa and PKAc account for the generation of endocrine tumors and chimeric fusions with PKAc have been linked to hepatocellular carcinoma^7^,^16^,^17^,^18^,^19^,^20^,^21^,^22^,^23^,^24^.

To detect and systematically map changes of PPIs involved in aberrant signal transmission, easily adaptable assays are needed. Such PPI reporters would be valuable for genetic or pharmacological studies in different cell- or organism-based model systems. In particular, the identification of upstream regulators, patient mutations, and/or specific molecules that affect PPIs are required to understand and target the pathological cell condition, specifically PPIs emanating from GTPases and kinases^4^,^16^,^25^,^26^. A simple PPI reporter assay that can be used in different model systems would be convenient for drug discovery in several respects. Besides the quantification of selected wild type and mutationally modified protein complexes, the same PPIs could serve as reporter and quantitative read-out for upstream enzyme or receptor activities.

However, time-resolved quantification of dynamic PPI in living cells and across model organisms is often limited due to the complexity of the biosensor systems needed to study them. Existing methods, such as those based on fluorescence and bioluminescence resonance energy transfer (FRET, BRET) are technologically challenging, they have a narrow dynamic range, and the interpretation of the results of such experiments is demanding, especially in living subjects^27^,^28^,^29^,^30^.

We have advanced a highly specific *Renilla* Luciferase (*R*luc) Protein-fragment Complementation Assay (PCA)^31^,^32^,^33^,^34^,^35^ to systematically map explicit features of defined PPIs *in vivo*. We show that precise and time-dependent quantifications of dynamic PPIs are easily adaptable to applications in any cell, tissue, or organism. Moreover, we illustrate that an advanced PPI reporter platform based on mutually exclusive binary interactions of PKA can be applied to analyze PPI dynamics following activation of endogenous GPCR pathways in different model systems.

## Results

**Figure 1:**
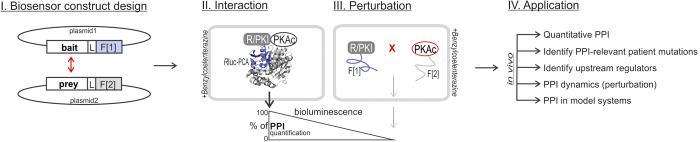
Biosensor design principle. Design principle and features of the *R*luc PCA: Indicated PKA subunits have been tagged with *R*luc PCA fragments (F[1], F[2]; L, 10 aa linker). PPIs trigger folding and reconstituted activity of appended PCA-fragments. The emitted luminescence signal is a quantitative reporter of cellular PPIs. Perturbations (indicated with X) through small molecules, upstream factors, competitive PPIs, or mutations reduce protein complex formation. A selection of applications of the PPI reporter are indicated.

Biosensor design and preferable features of the *R*luc PCA-based PPI reporter are shown in Fig. 1. Genetically encoded *R*luc PCAs are applied to quantification and characterization of dynamic PPIs in real time and *in vivo*. The impact of perturbations of binary PPIs and upstream factors can be determined quantitatively. Specifications of PPI dynamics offer the possibility to be used for functional cellular read outs, for example of oncogenic PPI and receptor-effector interactions. It is desirable to quantify differential PPIs in distinct eukaryotic model systems to investigate physiological and pathological cell states.

**Figure 2:**
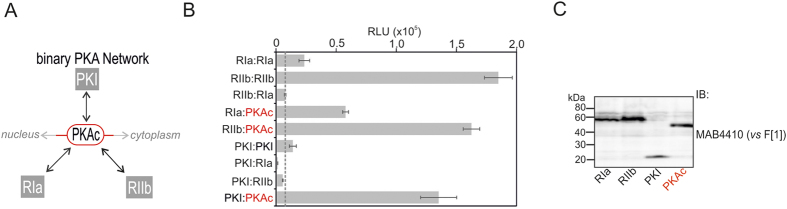
PPI analyses of mutually exclusive binary PKA interactions. (**A**) Schematic depiction of compartmentalized PPIs of the cAMP-controlled PKA network. Besides alterations of cAMP levels, mutations in PKAc and RIa affect PKAc localizations and phosphotransferase activities. (**B**) Co-transfection of HEK293 cells with indicated *R*luc PCA pairs followed by *R*luc PCA analyses have been performed. The amount of PKI hybrid constructs have been bisected for cell transfections (representative of n = 3; SEM from triplicates). (**c**) Immunoblotting of F[1]-tagged PCA hybrid proteins have been performed with Rluc F[1]-specific monoclonal antibodies (Millipore, #MAB4410); representative experiment.

Here we tested the impact of distinct perturbations of proteins and/or receptor pathways (GPCRs) on defined molecular interactions using an extended PPI reporter platform. We analyzed unique properties of oncogenic bait-protein interactions with exchangeable prey proteins in cell culture and in diverse model organisms. For this purpose we constructed new *R*luc PCA pairs to assess differential PPI. We selected PPI pairs of the binary and compartmentalized PKA network (Fig. 2A).

The first step was generation and benchmarking of highly selective *R*luc PCA pairs from the dynamic PKA network, which we subjected to cell and organism-based perturbation studies (Fig. 2A). Originally we reported the engineering of the *R*luc PCA PPI reporter consisting of PKAc and cAMP-sensing RIIb subunits. In the initial study we showed quantifications of PPI dynamics in different cell lines^33^. To understand the mechanism of complex formation of PKAc we generated new PCA pairs with differentially localized phosphotransferase inhibitors, such as RIa and PKI (Fig. 2B). GPCR-controlled cAMP-mobilization is sensed by differentially localized R subunits bound to PKAc. cAMP-binding to R leads to dissociation and activation of PKAc^12^. In addition to cAMP-degradation and re-association of the PKA holoenzyme^12^,^36^, PKI binding to nuclear PKAc represents another mechanism of A-kinase phosphotransferase inhibition^15^ (Fig. 2A). Following C-terminal tagging with *R*luc PCA fragments we compared dimerization of homo- and heterodimers of PKA. Genetically encoded bait-F[1] and prey-F[2] hybrid-constructs were transiently over-expressed in HEK293 cells. Besides RIa and RIIb homodimers, we observed complex formation of heterodimers of PKAc with RIa, RIIb and PKI. We assume that the low dimerization signals of RIa:RIa compared to RIIb:RIIb originate from differences in the tetrameric RIIb:PKAc and RIa:PKAc holoenzyme structures^12^ and thus suboptimal orientation of the *R*luc PCA fragments for complementation. No significant PPIs of R subunits heterodimers and R:PKI interactions were evident (Fig. 2B). The dotted line is aligned with the signal for co-expressed RIa and RIIb *R*luc PCA constructs indicating the arbitrary background signal. Comparable expression levels of the hybrid proteins tagged with *R*luc F[1] were observed (Fig. 2C).

The prime targets for pharmaceutical intervention are membrane receptors, and GPCRs are the largest superfamily of cell surface molecules involved in signal transmission. A large portion of these GPCRs couple to cAMP-mobilization^9^,^11^,^37^. We set out to activate defined endogenous receptor cascades first in defined cell settings and then in various model organisms.

**Figure 3:**
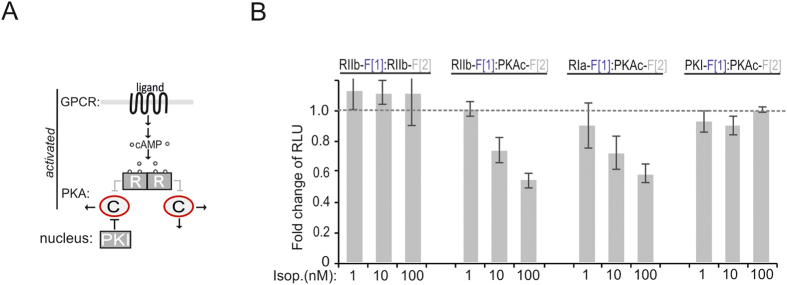
Impact of receptor activities on PPIs of PKA in human osteosarcoma cells. (**A**) Ligand-mediated activations of GPCR pathways linked to cAMP production lead to activation of PKA holoenzymes. cAMP binds to R subunits and triggers dissociation of active PKAc subunits which phosphorylate substrates in cytoplasm and nucleus. PKI inactivates nuclear PKAc (C) complexes. (**B**) U2OS cells transiently expressing indicated *R*luc PCA pairs were exposed to different doses of isoproterenol (15 min). PPIs were determined using the *R*luc PCA as read out (normalized to the untreated control for each PPI reporter; n = 3 independent experiments; SEM).

The prototypical GPCR coupled to cAMP production is the beta adrenergic receptor family (βAR). Activation of different βAR subtypes are related to proliferation, cardiac function, and memory and learning^37^,^38^,^39^,^40^,^41^. We chose the human osteosarcoma cell line U2OS (which exclusively expresses β_2_ARs) for the first perturbation experiments of cellular cAMP-levels^33^. We tested how β_2_AR-mediated cAMP-production affects binary PPIs of the PKA network (Fig. 3A). We activated endogenous β_2_AR in U2OS cells with the non-selective beta adrenergic agonist isoproterenol. Following overexpression of indicated *R*luc PCA pairs we observed that nM concentrations of isoproterenol are sufficient to induce type I and type II PKA activation, indicated by a decrease of PPI/bioluminescence. Activation of β_2_ARs for 15 min has no impact on the RII dimer and on PKAc:PKI interactions (Fig. 3B).

**Figure 4:**
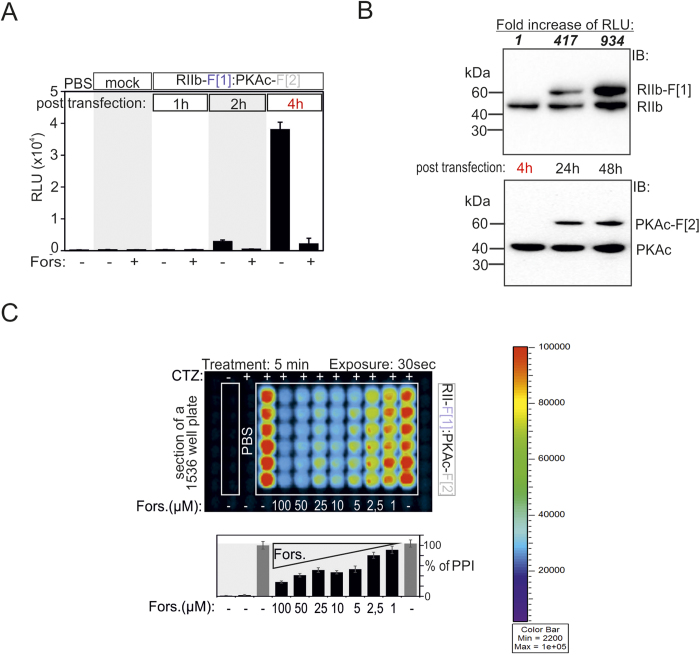
Analyses of PKA reporter sensitivity and its miniaturization using human cells. (**a**) Time-dependent evaluation of complex formation of RIIb-F[1]:PKAc-F[2] hours post transfection (1 h, 2 h and 4 h). The effect of forskolin (50 μM; 10 min) on PKA complex formation has been determined. (**b**) Fold increase of luminescence signals originating from the complemented *R*luc PCA based PKA reporter following transient overexpression in HEK293 cells for indicated time-frames is shown. Immunoblotting shows expression levels of endogenous and overexpressed PKA subunits. The same membrane has been probed first with RIIb and then with PKAc antibodies (BD Biosciences; #610626, #610981). (**c**) Dose-dependent effects of forskolin exposure on PPI-mediated luminescence measured simultaneously in 1536-well plate format. HEK293 cells transiently expressing the PKA reporter were treated for 5 min with 50 μM forskolin, transferred to a 1536 well plate and luminescence in the presence of benzyl-coelenterazine (CTZ) was captured for 30 sec on the Fusion imaging platform (Biorad). The pseudo-color scale indicates intensities of emitted luminescence signals. The quantification summarizes the effect of forskolin exposure on RIIb:PKAc interaction.

Following analyses of PKA dynamics upon activation of G-alpha-s coupled receptor cascades we benchmarked features of the *R*luc PCA, first in intact cells and subsequently in model organisms. We decided to focus our efforts on cytoplasmatic localized RIIb:PKAc interactions^33^. Genetically encoded RIIb-F[1] and PKAc-F[2] hybrid-constructs were transiently over-expressed in HEK293 cells. Within two hours of transfection we detected luminescence resulting from the PPI reporter. Addition of forskolin triggered dissociation of the PKA complex, which resulted in a disruption of the RIIb:PKAc complex and spontaneous unfolding of the *R*luc PCA reporter, and thus a decreased luminescence signal (Fig. 4A). Next, we quantitatively measured PPIs in a time-dependent manner. Four hours of transient over-expression of the reporter was sufficient to detect dynamic PPIs (Fig. 4A). However, at this stage, expression of the *R*luc PCA could not be tracked using immuno-blotting (Fig. 4B). Six-fold prolongation of the expression-time caused a 417-fold increase of the PPI signal (24 h). Consequently, quantification of PKA *R*luc PCA reporter (henceforth, the PKA reporter) was possible at varying expression levels of the reporter, when compared with those of the endogenous proteins, RIIb and PKAc respectively (Fig. 4B). Next we performed quantitative, dose-dependent measurements of PPIs with intact HEK293 cells in a 1536-well plate format to show that we could adapt this system for high throughput screenings (HTS). We illustrate that the simplicity and sensitivity of the *R*luc PCA allows simultaneous recordings of dose-dependent effects of forskolin on the PKA reporter in a feasible short time-frame (less than 10 min) for treatment and measurements (Fig. 4C).

**Figure 5:**
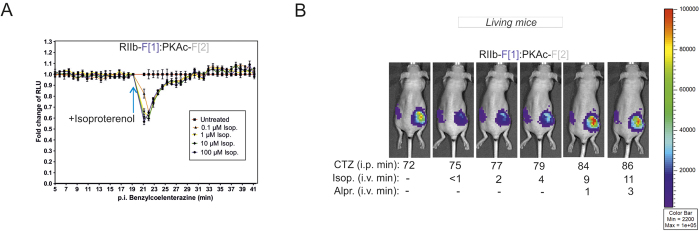
Benchmarking of PKA reporter dynamics in mouse xenografts. (**A**) Time- and dose-dependent effects of isoproterenol treatments on complex formation of RIIb-F[1]:PKAc-F[2] is shown (HEK293 cells stably expressing the *R*luc PCA PKA reporter). Luminescent signals from each well were normalized to the last point immediately preceding the administration of isoproterenol. (**B**) Subcutaneously engrafted HEK293 cells stably expressing the *R*luc PCA PKA reporter formed human tumor xenografts in living mice. Shown is time-dependent and non-invasive *in vivo* luminescence imaging of the PKA reporter in response to application of native coelenterazine (1.2 mg/kg; intra peritoneal, [i.p.]), isoproterenol (2.4 mg/kg mouse; intra venous [i.v.]) and alprenolol (8.0 mg/kg; [i.v.]). The pseudo-color scale indicates intensities of emitted luminescence signals.

We then used the *R*luc PCA PKA assay as a reporter to measure activation of distinct GPCRs. For this we employed HEK293 cells that express low levels of endogenous β_2_ARs^38^,^42^. Using a reporter cell line with stable expression of the PKA biosensor we recorded a transient dissociation of the RII:PKAc complex (within a 10 minute time interval) upon treatment with isoproterenol (Fig. 5A).

We next tested the PKA reporter in xenografts of living mice^43^. Genetically modified HEK293 cells stably expressing the *R*luc PCA-based PKA reporter were subcutaneously engrafted into host mice. Non-invasive imaging *in vivo* of PKA biosensor-generated luminescence from the tumor xenografts indicated formation of PKA complexes under basal conditions. We tested application of the PKA reporter to analyze endogenous GPCR activity. We recorded GPCR-triggered PKA activities (in time- and dose-dependent manner) following intra-venous (i.v.) administration of isoproterenol and the βAR antagonist alprenolol, and showed that the dynamic PKA reporter immediately responded to elevations in endogenous βAR activities. Time-resolved imaging of PKA activity further illustrated that βAR activity could be reversed by subsequent treatment with the non-selective βAR antagonist alprenolol (Fig. 5B). In comparison, administration of 0.9% saline showed no significant impact on PKA activation, (*P* *=* 0,0014; Supplementary Figure S1). To exclude the possibility that isoproterenol might directly affect the luminescent signal output, we tested HEK293 xenografts expressing the full length *R*luc. I.v. administration of isoproterenol did not cause a decrease in *R*luc-mediated luminescent signals (Supplementary Figure S2). These experiments demonstrate that the compounds injected into the blood stream of the mouse reached the xenograft and affected the PKA reporter through modulation of endogenous GPCRs in measureable time-intervals of minutes.

**Figure 6:**
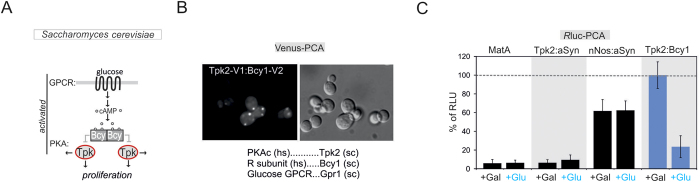
Benchmarking of PKA reporter dynamics in budding yeast. (**A**) Glucose-mediated activations of the *S.cerevisiae* GPCR Gpr1 leads to cAMP-production and PKA activation. cAMP binds to Bcy1 (R subunit) and triggers dissociation of active Tpk1-3 (PKAc subunits) which phosphorylate substrates and enhance yeast proliferation. (**B**) Localization of PKA in *S.cerevisiae* using overexpressed and Venus-PCA fragment-tagged yeast PKA subunits (Bcy1-V[1]:Tpk2-V[2]; V stands for Venus-PCA fragments). (**C**) Impact of indicated nutrient sugar sources (glucose and galactose) on complex formation of indicated *R*luc PCA fused protein pairs (Tpk2-F[1]:aSyn-F[2]; nNos-F[1]:aSyn-F[2]; Tpk2-F[1]:Bcy1-F[2]; SD from 3 independent experiments).

To demonstrate its broad applicability, we applied the PKA reporter to two other widely used model systems. We first analyzed the suitability of a simple but genetically well defined eukaryote, the budding yeast *Saccharomyces cerevisiae*. Studies of dynamic PPIs using enzymatic biosensors are limited in these cells due to their thick cell wall and low expression levels of PPI reporters. We chose *S*. cerevisae for two reasons: first it contains endogenous GPCR pathways (e.g. the glucose sensing Gpr1); second, GPCR pathways in budding yeast are linked to cAMP-elevation and PKA activation. PKA is highly conserved and in the yeast is composed of one regulatory (Bcy1) and three paralogous catalytic subunits (Tpk1-3)^44^,^45^ (Fig. 6A). High conservation at the protein level prompted us to try the same tagging strategy as for the mammalian *R*luc PCA based PKA sensor to generate a congruent yeast reporter system (Supplementary Figure S3). In parallel, we also generated the Venus-YFP PCA based PKA reporter to localize PKA complexes in yeast^35^,^46^. In Fig. 6B we show the subcellular localization of Venus-PCA tagged yeast PKA subunits (Tpk2-V[1]:Bcy1-V[2]) in budding yeast with a localized punctate pattern. We also over-expressed several *R*luc PCA based hybrid proteins. As a positive control for detection of PPI, we expressed *R*luc PCA tagged PDZ domains of neuronal nitric oxide synthase (nNOS) and aSyntrophin (aSyn), that are exogenous to yeast and known to form heterodimers^47^,^48^. Over-expression of *R*luc PCA fragment tagged nNOS:aSyn and Tpk2:Bcy1 showed a significant luminescence signal in comparison to indicated controls and the wild type *MAT***a** yeast strain without any PCA fragment. Next, we treated cells with glucose following growth in galactose to activate the GPCR glucose sensor Gpr1. In comparison with the galactose control experiment, we observed that activation of Gpr1 through glucose triggered activation of the *R*luc PCA based PKA reporter (Fig. 6C). These data underline the suitability of the *R*luc PCA for studying dynamic PPIs in budding yeast.

**Figure 7:**
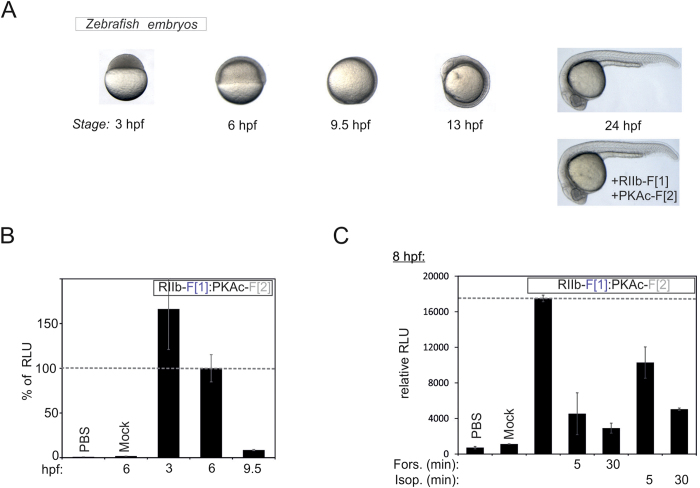
Benchmarking of PKA reporter dynamics in zebrafish embryos. (**A**) Illustration of developmental stages of fertilized zebrafish embryos with specification of elapsed hours post fertilization (hpf). Morphological analyses of reporter construct injection at 24 hpf. (**B**) Normalized quantification of PPI of RIIb-F[1]:PKAc-F[2] 3 hpf, 6 hpf and 9.5 hpf (zebrafish embryos; representative experiment). (**C**) Impact of time-dependent forskolin (50 μM) and isoproterenol (10 μM) exposure of de-chorionated zebrafish embryos on complex formation of overexpressed *R*luc PCA based PKA reporter at the 8 hpf stage (representative of n = 3; SEM from independent measurements).

Finally, we tested the *R*luc PCA PKA reporter in zebrafish (*Danio* rerio) embryos, a widely used vertebrate developmental model system, which has recently also been adapted for drug discovery purposes, particularly to screen for molecules that act on the common GPCR family of receptors 10,11,49,50,51,52,. GPCR pathways play critical roles at different stages of development (e.g. Wnt signaling, Hedgehog, and chemokine signaling)^9^,^10^,^53^,^54^,^55^. We attempted to determine if embryos respond to external activation of βARs and if direct activation of adenylyl cyclases (ACs) is detectable using our reporter system. Over the course of a 24 h time frame, a variety of developmental stages of fertilized zebrafish embryos can be distinguished (Fig. 7A). First, we showed that injection of equal amounts of mRNA encoding the *R*luc PCA-based PKA reporter constructs (RII-F[1]:PKAc-F[2]) did not affect embryo development by morphological criteria (Fig. 7A). We focused on stages between 3 and 24 hours post fertilization (hpf). After removing the chorion, embryos were subjected to *R*luc PCA measurements. We observed PKA originating *R*luc PCA signal decrease with time (Fig. 7B). After removing the chorion we treated embryos either with forskolin or isoproterenol for five and 30 min followed by *R*luc PCA analyses. In comparison to the controls, we observed changes in PKA reporter signal. Both forskolin and isoproterenol treatment triggered PKA activation in the embryos at the 8 hpf stage (Fig. 7C). Also after 24 h hpf it was possible to analyze dynamic PPIs (Supplementary Figure S4). These data indicate that for elaborated PPI studies in the zebrafish model system it will be necessary to generate transgenic animals. These experiments indicate that *R*luc PCA-based reporters are suitable for analysis of dynamic PPIs at different embryological stages of zebrafish embryos. This can be also useful for real-time recordings of defined receptor-regulated PPIs in and between developmental stages using either wild type or specific disease-relevant mutant strains. The PKA activity status is controlled through a variety of cAMP linked GPCRs which are prime targets for drug discovery^10^,^11^,^39^,^53^,^54^,^55^,^56^. Therefore the PKA reporter can be adapted to studies on pharmaceutically relevant and endogenously expressed GPCRs in the zebrafish model. It is therefore suitable to test small-molecules acting on GPCRs in this model through both Gαs- and Gαi-coupled receptors that exist in zebrafish. Several small molecules act on these receptors and their discovery have resulted in preclinical and clinical trials^10^. One major advantage of the zebrafish embryos is that the efficacies of small molecules targeting PKA-linked pathways can be assessed. Simultaneously a phenotypic toxicology profile can be applied, potentially accelerating drug discovery.

## Discussion

In this study, we have illustrated the general utility of the *R*luc PCA reporter to study PPI dynamics in selected model organisms that are used in current biomedical research.

The simplicity and sensitivity of the PPI reporter enables systematic protein complex quantifications in real time, using a simple and straight forward protocol. The PKA reporter is functional at expression levels far below endogenous protein expression levels (the Rluc PCA was not detectable in the immunoblotting experiment, Fig. 4B), due to extremely high signal to background levels. This is desirable for studies on model organisms because it reduces the potential impact of ectopic protein expression on cell homeostasis. Dynamics of the studied PPIs reflect linkage to upstream receptor pathways. In the context of drug efficacies and timing, the PPI reporter allows for accurate and quantitative specification of receptor-effector relationships directly in the preferred cell type or model organism. The design of further PCA-based biosensors at different stages within cascades and between critical hubs of lead molecules signal transmission will facilitate the identification of signaling crosstalk and related OFF-target effects of lead molecules^57^. Existing PPI reporter based on FRET or BRET^58^,^59^,^60^ are technologically challenging to implement. We show that with a simple *R*luc PCA protocol PPI quantification of cell populations in high content format is possible with standard lab equipment. Moreover, we illustrate that the *R*luc PCA is a robust readout for PPI even at expression levels far below the endogenous bait and prey proteins. It is the high signal-to background ratio which permits a more accurate detection of PPI dynamics in different *in vivo* settings. However, structural information is helpful to generate a functional *R*luc PCA reporter. For BRET and FRET reporter, the distance between the bait and prey and their orientation is critical for energy transfer to take place. For the PCA reporter it is more restrictive, the fragments need to be brought into close proximity to allow complementation of functional reporter activity. Without knowledge of the binding domains, the size of bait and prey proteins is the critical factor for *R*luc PCA design^33^,^35^. Referring to this, knowledge of the structural constraints of PPI interfaces is helpful to construct a functional reporter. In addition, the luminescence signal lifetime and low photon emission rates limit the applicability of the Rluc PCA reporter for single cell studies.

Taking these aspects of *R*luc PCA reporter design and measurements into account, we generated a *R*luc PCA platform for analyses of mutually exclusive PPIs of the PKA network. In light of disease relevant RIa and PKAc variations (mutations, fusions, decontrolled upstream pathways)^7^,^17^,^18^,^19^,^20^,^21^,^22^,^23^ application of the PKA *R*luc PCA platform offers the possibility to systematically test different means of kinase perturbations using the available biosensor toolbox. We quantified mutually exclusive PKAc interactions which account for different spatially controlled cell functions.

We also highlight that *R*luc PCA analyses of dynamic PPIs are not restricted to specific cell types. We recorded PPI dynamics in three different model organisms, and envision the development of more wide-ranging intracellular reporter platforms. One remarkable finding of this study was the acquisition of real-time recordings of receptor-controlled PPI dynamics in xenografts of living mice. We show that even i.v. administration of counteracting small molecules, which act on endogenously existing GPCRs, show an immediate (time frame of minutes) and opposing impact on PPIs in the xenografts. In addition, we tested PPIs in budding yeast and zebrafish embryos. We showed that in zebrafish embryos, endogenous GPCR activities can be tracked over time, which extends the application spectrum for drug discovery. This long time-frame detection would allow for both testing of lead compound targeting with simultaneous applications of phenotypic toxicity tests.

In several studies, we have elaborated possible applications of the *R*luc-based PCA for defined functional read-outs in cell culture systems^45^,^61^,^62^,^63^,^64^,^65^,^66^,^67^,^68^. Now, such PPI-encoded functions can be directly quantified and recorded in the model organism of choice. In addition to measurements of GPCR, kinase and phosphodiesterase activities, PKA-related ubiquitination assays could to be feasible^33^,^62^,^63^. The presented *R*luc PCA based biosensor platform opens new perspectives to analyze the impact of environmental conditions and lead compounds sensed by membrane receptors on intracellular PPI networks. Here we show that this can be analyzed directly in the favorable model organism, which is a suitable new way to study signaling cross-talk and to quantify drug efficacies in a time-dependent manner.

## Materials and Methods

### *Renilla* luciferase PCA

*Constructs:* The *R*luc PCA based hybrid proteins RIIb–F[1] and PKAc-F[2] have been designed as previously described^33^. *R*luc PCA fusions with PKI and RIa have been generated using an analogous cloning approach. Following PCR amplification of human RIa (alpha) (protein accession number: NP_002725.1) and PKI alpha (protein accession number: AAA72716; addgene plasmid # 45066)^69^ we fused them C-terminally with either F[1] or F[2] of the *R*luc PCA. *PPI measurements*: HEK293 and U2OS cells were grown in DMEM supplemented with 10% FBS. We transiently overexpressed indicated versions of the *R*luc PCA based biosensor in 24 or 12 well plate formats. One, 2,4 ,24 or 48 hours post-transfection we exchanged growth medium and resuspended cells in PBS. We scraped down the cells in case of the U2OS cells. Cell suspensions were transferred to 96-well plates and subjected to luminescence analysis using the LMax^TM^-II-384 luminometer (Molecular Devices). *R*luc luminescence signals were integrated for 10 seconds following addition of the *R*luc substrate benzyl-coelenterazine (5 μM; Nanolight)^33^. Dose-dependent effects of forskolin exposure on PPI-mediated luminescence signals were measured simultaneously in 1536-well plate format. Detached HEK293 cells transiently expressing the PKA reporter were treated for 5 min with increasing concentrations of forskolin in 1.5 ml test tubes. Immediately after forskolin exposure (5 min) aliquots of cells (+5 μM benzyl-coelenterazine) were transferred to a 1536 well plate and the luminescence was captured for 30 sec on the Fusion imaging platform (Biorad).

### Yeast *R*luc-PCA reporter construction

All constructs are plasmid based and candidate genes are expressed in fusion with either Venus or *R*luc PCA fragments under constitutive ADH or TEF promoter. To create the constructs, *TPK2* and *BCY1* gene open reading frames were PCR amplified from yeast genomic DNA and respectively cloned into the plasmids p413-linker-VenusF[1], p415-linker-VenusF[2] (for Venus PCA) and p413-linker-*R*lucF[1], p415-linker-*R*lucF[2] (for *R*luc PCA) using SpeI and BamHI restriction enzymes 5’ to the linker. As positive control for detection of PPI, nNOS and aSyn PDZ domain DNA sequences were PCR-amplified from template DNAs (a kind gift from W.A. Lim, UC San Francisco) and cloned into p41NAT-linker-*R*lucF[1] and p41HPH-linker-RlucF[2] plasmids, respectively, between XbaI and BspEI sites located 5’ to the linker. PDZ-linker-*R*luc PCA fragment fusions were expressed from a plasmid under the control of the TEF promoter. These result in plasmids p41NAT-nNOS-RlucF[1] and pHPH-aSyn-RlucF[2], respectively^47^.

### Yeast PCA reporter measurements

Plasmids containing the appropriate candidate genes were either singly or co-transformed into competent MATa haploid yeast cells using the standard Lithium acetate method. Positive clones were selected on the synthetic complete media lacking Histidine (His) (p413 constructs) and Leucine (Leu) (p415 constructs) either as single or double drop out selection. The nNOS and aSyn positive clones were selected using either Clonat or Hygromycin antibiotic resistance, respectively. Single positive clones were grown overnight in low fluorescent media containing galactose to make a pre-culture. From the pre-culture, cells were diluted into fresh media at 0.05OD_600_ and continued to grow in low florescent galactose media until 0.1OD_600_. To detect the Tpk2 interaction with Bcy1 using Venus PCA cells grown in low florescent galactose media were plated onto black multi-well plates precoated with Concanavalin A, allowed to settle for 10 minutes and imaged using a Nikon Eclipse TE2000U inverted microscope (Nikon) with 60X oil objective and DIC or YFP filter cube (41028, Chroma Technologies). Images were captured with a CoolSnap CCD camera (Photometrics) using MetaMorph software (Molecular Devices). The PPI signals using *R*luc PCA were measured using cells equivalent to 0.1OD_600_ (approximately 1 × 10^6^ cells). Cells were grown in low fluorescent media with appropriate selection. For each sample, cells equivalent to 0.1OD_600_ were spun, supernatant was discarded and cells were re-suspended in 160 μl of fresh medium. Cells were transferred to white 96-well flat bottom plates. The Luciferase substrate benzyl-coelenterazine (Nanolight #301) was diluted from the stock (2 mM in absolute ethanol) using 1x phosphate-buffered saline (PBS), pH7.2, containing 1 mM EDTA. Glucose was diluted to 1 μM from 2 M stock using 1x PBS. LMax II384 Luminometer (Molecular Devices, Sunnyvale, CA, USA) was used to measure the *R*luc PCA signal. Using the internal injectors of the luminometer, 20 μl each of diluted benzyl-coelenterazine (to a final concentration of 10 μM) and glucose (to a final concentration of 100 mM) were added to the cell mixture, mixed by shaking and incubated for 60 seconds. After incubation, the *R*luc PCA signal was integrated for 30 seconds. In a single experiment, for each sample the signal was measured in triplicates and in total, experiments were repeated independently three times^47^.

### Mouse xenografts

1 × 10^7^ HEK293 cells stably co-expressing the PCA hybrid proteins RII-F[1] and PKAc-F[2] were injected subcutaneously into the right flank of 9–10 week-old female athymic NCr-nu/nu mice (NCI Frederick). After 16 days, animals were imaged by the IVIS 200 system (Perkin Elmer). From 10 min after the intra-peritoneal (i.p.) injection of 1.2 mg/kg native coelenterazine, the animals were serially imaged under 2.0–2.5% isoflurane anesthesia every 2–5 min. When luminescence signal reached plateau, the animals were injected intravenously with 2.4 mg/kg isoproterenol. 7–8 min later the animals were injected intravenously with 8.0 mg/kg alprenolol. All mouse animal studies were performed in accordance with protocols approved by the Johns Hopkins Animal Care and Use Committee.

### Zebrafish embryos

RIIb-F[1] and PKAc-F[2] mRNA was generated using the mMESSAGE mMACHINE T7 Transcription Kit (Life Technologies), and 200 pg of each mRNA was microinjected into one cell stage zebrafish embryos. Embryos were raised at 28 °C until 3, 6, 8, 9.5 or 24 hpf, dechorionated and subjected to pharmacological treatments (Forskolin, 50 μM; Isoproterenol, 10 μM) at 28 °C for five and 30 minutes. Immediately afterwards, we resuspended the embryos and subjected the intact cells to luminescence measurements. All zebrafish experimental protocols were approved by the Austrian Ministry for Science and Research (GZ BMWF-66.008/0019-II/3b/2013), and experiments were carried out in accordance with approved guidelines

## Additional Information

**How to cite this article**: Röck, R. *et al* *In-vivo* detection of binary PKA network interactions upon activation of endogenous GPCRs. *Sci. Rep* **5**, 11133; doi: 10.1038/srep11133 (2015).

## Supplementary Material

Supplementary Information
