# Supplementary material for: In-vivo detection of binary PKA network interactions upon activation of endogenous GPCRs
Source: Sci Rep. 2015 Jun 23;5:11133. doi: 10.1038/srep11133 (PMC4477410; doi:10.1038/srep11133)
Supplement: Supplementary Information [file srep11133-s1.doc]

***Supplementary Information***

*In-vivo recordings of binary PKA network interactions upon activation of endogenous GPCRs*

Running title: Quantification of dynamic protein-protein interactions *in vivo*

Ruth Röck^1#^, Verena Bachmann^1#^, Hyo-eun C Bhang^2,*^, Mohan Malleshaiah^3,§^, Philipp Raffeiner^1^, Johanna E Mayrhofer^1^, Philipp M Tschaikner^4^, Klaus Bister^1^, Pia Aanstad^4^, Martin G Pomper^2^, Stephen W Michnick^3^, Eduard Stefan^1^

^1^ Institute of Biochemistry and Center for Molecular Biosciences, University of Innsbruck, Innrain 80/82, 6020 Innsbruck, Austria

^2^ Russell H. Morgan Department of Radiology and Radiological Science, Johns Hopkins Medical School, Baltimore, MD 21287, USA

^3^ Département de Biochimie, Université de Montréal, H3C 3J7 Montréal, Québec, Canada

^4^ Institute of Molecular Biology, University of Innsbruck, Technikerstrasse 25, 6020 Innsbruck, Austria

^#^ Equal contribution

^*^ Current Address: Department of Oncology, Novartis Institutes for Biomedical Research, Cambridge, MA 02139, USA

^§^ Current Address: Department of Systems Biology, Harvard Medical School, Boston, MA 02115, USA.

Correspondence should be addressed to Eduard Stefan (eduard.stefan@uibk.ac.at)
